# Cyberbullying Among School Adolescents in an Urban Setting of a Developing Country: Experience, Coping Strategies, and Mediating Effects of Different Support on Psychological Well-Being

**DOI:** 10.3389/fpsyg.2021.661919

**Published:** 2021-04-09

**Authors:** Anh Toan Ngo, Anh Quynh Tran, Bach Xuan Tran, Long Hoang Nguyen, Men Thi Hoang, Trang Huyen Thi Nguyen, Linh Phuong Doan, Giang Thu Vu, Tu Huu Nguyen, Hoa Thi Do, Carl A. Latkin, Roger C. M. Ho, Cyrus S. H. Ho

**Affiliations:** ^1^Institute for Preventive Medicine and Public Health, Hanoi Medical University, Hanoi, Vietnam; ^2^Bloomberg School of Public Health, Johns Hopkins University, Baltimore, MD, United States; ^3^Department of Global Public Health, Karolinska Institutet, Stockholm, Sweden; ^4^Institute for Global Health Innovations, Duy Tan University, Da Nang, Vietnam; ^5^Faculty of Medicine, Duy Tan University, Da Nang, Vietnam; ^6^Institute of Health Economics and Technology, Hanoi, Vietnam; ^7^Center of Excellence in Evidence-Based Medicine, Nguyen Tat Thanh University, Ho Chi Minh City, Vietnam; ^8^Vietnam Young Physician Association, Hanoi, Vietnam; ^9^Department of Psychological Medicine, Yong Loo Lin School of Medicine, National University of Singapore, Singapore, Singapore; ^10^Institute for Health Innovation and Technology (iHealthtech), National University of Singapore, Singapore, Singapore; ^11^Department of Psychological Medicine, National University Health System, Singapore, Singapore

**Keywords:** cyberbullying, social support, psychological health, structural equation modeling, adolescent

## Abstract

**Background**: This study examined the cyberbullying experience and coping manners of adolescents in urban Vietnam and explored the mediating effect of different support to the associations between cyberbullying and mental health issues.

**Methods**: A cross-sectional study was performed on 484 students at four secondary schools. Cyberbullying experience, coping strategies, psychological problems, and family, peer, and teacher support were obtained. Structural equation modeling was utilized to determine the mediating effects of different support on associations between cyberbullying and psychological problems.

**Results**: There were 11.6 and 28.3% of students who reported that they experienced and observed at least one cyberbullying act in the last 3 months, respectively. Among the victims, only 48.2% tried to stop the perpetrators. Meanwhile, the majority of observers belonged to the “Intervene” group who tried to report cyberbullying acts or help victims. Family support was found to partially mediate associations between cyberbullying experience and observation with levels of psychological problems among adolescents.

**Conclusion**: The 3-month rate of cyberbullying experience and observation among urban adolescents aged 11–14 was low. However, current coping strategies against cyberbullying were not sufficient. Family support is an important factor that should be considered for designing interventions to mitigating the impacts of cyberbullying on the mental health of adolescents.

## Introduction

Cyberbullying has been well-documented as a global public health problem. Cyberbullying includes acts such as posting publicly available information on the Internet, being called or receiving messages that threaten or being harassed on the Internet, and others (Patchin and Hinduja, 2006). Compared with traditional bullying, cyberbullying can occur every time and everywhere, and the identity of the perpetrator may not be disclosed (Patchin and Hinduja, 2006). Therefore, cyberbullying may be more frequent and have more serious consequences for the victim. Studies show that cyberbullying significantly affects adolescents, such as increasing the risk of depression, anxiety, and suicidal ideation (Klomek et al., 2010; Schneider et al., 2012; Nixon, 2014; Fahy et al., 2016; Pabian and Vandebosch, 2016; Yuchang et al., 2019), as well as causing physical and psychosomatic problems such as difficulty sleeping, headache, and loss of appetite (Beckman et al., 2012; Schneider et al., 2012; Schultze-Krumbholz et al., 2012; Kowalski and Limber, 2013). Cyberbullying is also associated with the onset of substance use, traditional and cyberbullying perpetration, and delinquency (Mitchell et al., 2007; Wong et al., 2014; Chan and Wong, 2020; Estévez et al., 2020). These consequences have been observed in all perpetrators, victims, and observers of cyberbullying (Beckman et al., 2012; Wong et al., 2014; Panumaporn et al., 2020).

Cyberbullying among adolescents is a prevalent phenomenon across nations. A prior systematic review showed that 20 to 40% of adolescents experienced cyberbullying at least once in their lifetime (Aboujaoude et al., 2015), and this rate tends to increase with increasing Internet and social media accessibility (Hamm et al., 2015). In the United States, the rate of adolescents who were victims of cyberbullying was from 3 to 72% (Selkie et al., 2016). A study conducted in seven European countries showed that 13.3–37.3% of adolescents aged 14–17 years were victims of cyberbullying (Athanasiou et al., 2018). In the Asian region, a review in Chinese populations revealed different prevalence of cyberbullying victimization in adolescents, ranging at 14–57% in mainland China, 13–35% in Taiwan, and 12–72% in Hong Kong (Chan and Wong, 2015). In Korea, 14.6% adolescents were cyberbullying victims (Lee and Shin, 2017). Coping strategies when having cyberbullying experience may vary and can be classified into four groups: (1) directly reacting against cyberbullying acts (such as retaliation or constructive feedback); (2) ignoring the cyberbullying behaviors (such as avoidance or doing nothing), (3) seeking support from other sources (such as parents, friends, or teachers), and (4) utilizing technological solutions (such as blocking senders; Perren et al., 2012). A study in Hong Kong indicated that older male adolescents were more likely to have an active approach to cope with cyberbullying such as informing to adults, parents, or teachers; while adolescents, having limited experience with their schools, tended to have avoid (e.g., ignore the cyberbullying behaviors) approaches (Chan and Wong, 2017). Another study in Czech adolescents found that technological strategies were the most common, following by avoidance and finding support (Machackova et al., 2013).

The proliferation of cyberbullying requires accelerating efforts to explore approaches to prevent and mitigate its consequences. Global studies showed that social support is an essential component in protecting adolescents from the consequences of traditional bullying (Kochenderfer-Ladd and Skinner, 2002; Davidson and Demaray, 2007; Rothon et al., 2011). Social support refers to instrumental support (such as providing the victim with helpful solutions or resources) or emotional support (such as spiritual encouragement, belongingness, or value recognition; House et al., 1988; Kerres Malecki and Kilpatrick Demaray, 2002). In literature, many studies have shown that family and friends support can play an important role in protecting adolescents from becoming victims of cyberbullying (Price and Dalgleish, 2010; Fanti et al., 2012; Hellfeldt et al., 2019). In addition, adolescents who are of school age can get help from teachers. Previous research has shown that victims and perpetrators of cyber-violence possibly knew each other at school, and parents, friends, and teachers are important sources of information and support for teens dealing with cyberbullying (Slonje and Smith, 2008).

Although many studies showed that social support helps to prevent cyberbullying among adolescents, its effectiveness in minimizing the psychological consequences of cyberbullying among victims or observers has been in debate. An earlier study found that support from friends alone was effective in reducing psychological problems among victims of cyberbullying (Holt and Espelage, 2007). Another research found that both support from family and friends played an important role in preventing victims from the mental consequences of cyberbullying (Rothon et al., 2011). These results concluded that social support held the potential for minimizing the cyberbullying-related psychological problems in youths. However, the evidence for this effect in different population groups (such as victims, perpetrators, or observers) is limited.

In Vietnam, evidence about cyberbullying among adolescents as well as the impact of social support on cyberbullying is currently limited. Only one previous study was conducted on 215 adolescents and youths aged 13–18 in Hanoi, Vietnam, to measure the cyberbullying experience in these groups (Chi et al., 2020). By using the modified Patchin and Hinduja’s scale, this study found that 45.1% of sample had experienced cyberbullying at least once, with being called by names as the most typical form (Chi et al., 2020). The common responses to cyberbullies included ignoring cyberbullying behaviors and not telling family or teacher (Chi et al., 2020). To date, none of the studies were conducted about the mediation effect of social support on mental disorders in adolescents who were victims or observers of cyberbullying. Therefore, our study was conducted to examine the experience of adolescents aged 11–14 in urban Vietnam, determine how they coped with this issue, and explore the preventive mediation effect of social support to the associations between cyberbullying and mental health issues.

## Materials and Methods

### Theoretical Framework

In this study, we employed stress buffering hypothesis (Cohen and Wills, 1985), which perceived social support that could mediate the relationship between stressors (i.e., cyberbullying in this case) and their negative consequences (i.e., mental problems). In other words, a higher level of social support could more weaken this relationship. Cohen and Wills in their study suggested that social support could diminish people’s perceptions about the threat of given stressors, or offer coping options or other necessary resources to individuals against the stressors (Cohen and Wills, 1985). *Via* literature review, we hypothesized that support from family, peers, and teachers might play a buffering role in mediating the effect of cyberbullying experience on the mental health of adolescents (Holt and Espelage, 2007; Rothon et al., 2011). Therefore, we examined the direct effect of cyberbullying experience on adolescents’ mental health, as well as the indirect effect of social support in buffering relationships between cyberbullying and mental health.

### Study Design

Data of this paper were collected through a cross-sectional study conducted in Hanoi, Vietnam, from January to September 2020. Four secondary schools were randomly selected in this study from a list of secondary schools in Hanoi. The school principal and teachers were approached by the research team and informed of the research content. The questionnaire used in this study was submitted to them for approval before implementing data collection.

Participants included students aged 11–14 years, attending four selected secondary schools; and they, as well as their parents and teachers, agreed to be enrolled in the study. This study used a formula to estimate a population proportion with specified relative precision to calculate the essential sample, with *p* = 0.45 (according to previous research in Hanoi, Vietnam; Chi et al., 2020); confidence level *α* = 0.05; relative precision *ɛ* = 0.2. The sample size needed for a school was 118 students or 472 students/4 schools. An additional 10% sample size was added to prevent participants from dropout or nonresponse, resulting in 520 students (or 130 students per school) being invited to respond to questions about cyberbullying.

A multi-stage sampling method was applied. First, the research team randomly selected two classes in each grade of each school, resulting in 32 classes being selected for the sample. Next, in these 32 classes, 520 students were randomly selected to participate in the cyberbullying survey. There were 36 students who did not agree to participate, leading to a total of 484 students (response rate of 93.1%). These students, along with their parents or guardians, were provided written informed consent with brief information about research objectives, eligible criteria, research process, and benefits and requirements during study participation. This research protocol has been approved by the institutional review board of Hanoi Medical University (Code 22NCS17/HDDDDHYHN).

### Data Collection and Measurement

Students participating in this study were asked to complete an anonymous survey questionnaire. The research team directly distributed the questionnaires to students. During the survey, parents, teachers, and unselected students did not present at the site of the survey to avoid their influence on the participants’ responses. Each student spent 15–20 min completing the questionnaire. A structured questionnaire was used for this study. The content of the questionnaire was developed under the guidance of child violence experts. The questionnaires were pretested on 10 adolescents to ensure understandability, expression, and logic, which aimed to avoid misunderstanding or confusion to study participants.

### Variables

#### Cyberbullying Experience/Bystander and Coping Strategies

In this study, we used the Cyberbullying Test instrument to identify the individual’s experience and observation of cyberbullying (Garaigordobil, 2017). Originally, this tool asked participants to answer 45 items about 15 cyberbullying acts in three roles (15 items per role): perpetrators, victims, and observers. Examples of items in the instrument included the following: “Have they ever sent you offensive and insulting messages by cellphone or Internet?” “Have you ever received offensive and insulting calls on your cellphone or by Internet (Skype …)?” “Have you ever been assaulted to tape the assault and hang it on the Internet?” Each question had four options about from 0 “never” to 3 “always.” In this study, we used only two parts of the instrument: for victims and observers (or bystanders). Moreover, in the pilot, we observed that it was difficult for our participants (i.e., secondary school students) to respond to the questions with these four options. Thus, we decided to ask them a series of yes/no questions to determine whether they experienced cyberbullying acts as victims and observers in the last 3 months. This recall duration was applied to minimize the potential recall bias. Participants were categorized into “Cyberbullying experience” or “Cyberbullying observation” if they reported “yes” for at least one cyberbullying act. The Cronbach alpha values of “Cyberbullying experience” and “Cyberbullying observation” items were 0.8830 and 0.8993, respectively.

In this study, for people experiencing cyberbullying, we asked them to recall the impacts of these cyberbullying acts, coping strategies against cyberbullying, supporters when facing cyberbullying, and reasons for not reporting cyberbullying experience. Meanwhile, for those ever observing cyberbullying acts, participants were asked to report their reactions toward these behaviors. These reactions were classified into three groups: “Intervene,” “Ignore,” and “Join in.”

- “Intervene” included (1) “oppose acts of cyberbullying,” (2) “try to help or comfort the victim,” and (3) “report online violence to people able to help the victim (e.g., teachers and parents).”
- “Ignore” included “leaving cyberspace.”
- “Join in” included “Encouraging cyberbullying behaviors” and “Enjoys cyberbullying acts, and wants to learn more, but does not participate or promote publicly.”

In addition, we asked them to explain the reasons if they did not report the observed cyberbullying acts. We also asked all participants to express their attitude toward cyberbullying by asking them a question: “What extend do you agree or disagree with cyberbullying?” The students rated their attitude with an 11-point Likert scale from 0 “Totally agree” to 10 “Total disagree.”

#### Psychological Problems

To evaluate the psychological problems, the Depression, Anxiety, and Stress Scale—21 Items (DASS-21) was utilized. This instrument included 21 items regarding depression (seven items, range score 0–21), anxiety (seven items, range score 0–21), and stress symptoms (seven items, range score 0–21) in the last 7 days (Le et al., 2017). Examples of items in the instrument included the following: “I found it hard to wind down” (stress domain), “I was aware of dryness of my mouth” (anxiety domain), and “I could not seem to experience any positive feeling at all” (depression domain; Lovibond and Lovibond, 1996). Participants responded to each item on a four-point Likert scale from 0 “Did not apply to me at all” to 3 “Applied to me very much or most of the time” (Le et al., 2017). A higher score in each part indicated a higher severity of this psychological problem. The Vietnamese version of this instrument had been validated elsewhere (Le et al., 2017). The Cronbach alpha of this instrument was 0.8523.

#### Social Support

Support from family (two items), peer (two items), and teacher (three items) was measured by using seven items, as below:

1. My parents do not understand me or care about my feelings.
2. My parents do not listen to me or do not pay attention to the problems I have.
3. My classmates are very friendly.
4. My classmates respect me and listen to my opinion.
5. My teachers help me when I’m sad or having problems.
6. My teachers take care of me and support me in achieving the best results.
7. My teachers respect me and listen to me.

Students rated each item on a five-point Likert scale from 1 “Totally disagree” to 5 “Totally agree.” The score of each domain was computed by dividing the total scores of items in this domain by the number of items. Scores of questions 1 and 2 were reversed before computing the score of parental support. The score of each domain was from 1 to 5, with a greater score meaning a higher level of support. The Cronbach alpha of this instrument was 0.8205.

#### Sociodemographic Characteristics

In this part, we collected data about age, gender (male/female), and type of family (nuclear/multi-generations/others).

### Statistical Analysis

A *p*-value of <0.05 was used to detect a statistical significance. Stata 16.0 software was used for analyzing data. A listwise deletion approach was applied to handle missing data. Descriptive statistics were performed for all variables of interest. Statistical tests including chi-square and Mann-Whitney tests were used to examine the difference between sociodemographic characteristics, social support, cyberbullying attitude, and psychological problems between cyberbullying experience/non-experience and cyberbullying observation/non-observation. Multivariate logistic regression models were used to identify the factors associated with cyberbullying experience (yes = 1/no = 0, model 1) and cyberbullying observation (yes = 1/no = 0, model 2). Independent variables included sociodemographic characteristics (age, gender, and types of the family), support from family/peer/teachers, cyberbullying attitude, cyberbullying experience (for model 2), and cyberbullying observation (for model 1). Moreover, among those ever observing cyberbullying acts in the last 3 months (*n* = 136), we performed the multivariate logistic regression models to determine factors that were related to “Intervene” (yes = 1/no = 0, model 3) or “Ignore” (yes = 1/no = 0, model 4) behaviors. Independent variables for models 3 and 4 included sociodemographic characteristics (age, gender, and types of the family), support from family/peer/teachers, cyberbullying attitude, and cyberbullying experience. We did not perform the regression analysis for the “Join in” outcome because only nine students had these behaviors, which might not be a large enough sample size for the analysis.

Finally, structural equation modeling (SEM) was used to examine the mediation effects of family, peer, and teacher support on the relationships between cyberbullying experience/observation and psychological problems. The roles of cyberbullying (cyberbullying experience and observation) were coded as binary variables (yes = 1, no = 0), while the depression, anxiety, and stress variables were treated as continuous variables. The mean- and variance-adjusted maximum likelihood test statistic (MLMV) was performed for the SEM, given its robustness for data with non-normal distribution (Maydeu-Olivares, 2017). Multiple goodness-of-fit indices, including the root-mean-square error of approximation (RMSEA), the comparative fit index (CFI), and the standardized root mean square residual (SRMR) were examined. RMSEA lower than 0.08, SRMR lower than 0.08, and CFI higher than 0.09 were considered acceptable model fits (Kline, 2015).

### Ethical Approval

Given highly sensitive information collected from adolescents, we performed the following actions to ensure the rights and benefits of participants. First, we provided an information package to students and their parents/guardians before the survey implementation. This package had detailed information about the purposes of the study, study designs, eligible criteria, rights, and benefits of study participants. It was also emphasized in the information package that the participation of students was voluntary and that relationships between the students and teachers/schools would not be affected in any way if they did not participate in the survey. No individual data were collected to protect students’ privacy; thus, it was impossible to re-identify the participants based on the current dataset. Students and their caregivers were also informed that they could skip any questions that they felt uncomfortable or they could withdraw from the study at any time. We offered the helpline in the information package for students who needed help to address cyberbullying-related issues. Contacts of the principal investigators and coordinators of this study were provided to answer all questions raised about the study.

## Results

The characteristics of participants are presented in Table 1. Among 484 secondary school students, the mean age was 12.6 (*SD* = 1.2) years. There were 11.6 and 28.3% of students reporting that they experienced and observed at least one cyberbullying act in the last 3 months, respectively. No difference was observed regarding gender, age, and type of family between those with and without cyberbullying experience (*p* > 0.05). Meanwhile, the rate of females in the cyberbullying observation group (68.6%) was significantly higher than that in the non-cyberbullying observation (56.2%). A significant age difference was also found between these two groups (*p* < 0.001).

**Table 1:** Cyberbullying experienced and observation according to sociodemographic levels of support and mental problems (*n* = 484).

Characteristics	Cyberbullying					
	Experienced			Observation		
	Yes	No	*p*-value	Yes	No	*p*-value
	*n* (%)	*n* (%)		*n* (%)	*n* (%)	
Total	56 (11.6)	428 (88.4)		137 (28.3)	347 (71.7)	
Gender						
Male	26 (46.4)	169 (39.5)	0.319	43 (31.4)	152 (43.8)	0.012
Female	30 (53.6)	259 (60.5)		94 (68.6)	195 (56.2)	
Age (years)						
11	13 (23.2)	133 (31.1)	0.499	16 (11.7)	130 (37.5)	<0.001
12	5 (8.9)	50 (11.7)		16 (11.7)	39 (11.2)	
13	21 (37.5)	130 (30.4)		57 (41.6)	94 (27.1)	
14	17 (30.4)	115 (26.9)		48 (35.0)	84 (24.2)	
Type of family						
Nuclear	30 (53.6)	279 (65.2)	0.220	82 (59.9)	227 (65.4)	0.513
Multiple generations	25 (44.6)	141 (32.9)		52 (38.0)	114 (32.9)	
Others	1 (1.8)	8 (1.9)		3 (2.2)	6 (1.7)	
	Mean (*SD*)	Mean (*SD*)	*p*-value	Mean (*SD*)	Mean (*SD*)	*p*-value
Age (years)	12.8 (1.1)	12.5 (1.2)	0.213	13.0 (1.0)	12.4 (1.2)	<0.001
Cyberbullying attitude (1–10)	9.2 (2.2)	9.7 (1.5)	0.022	9.6 (1.6)	9.6 (1.6)	0.211
Support from family (1–5)	3.5 (1.1)	4.0 (1.0)	<0.001	3.7 (1.0)	4.0 (1.1)	<0.001
Support from peer (1–5)	2.4 (1.1)	2.0 (1.0)	0.005	2.2 (1.0)	2.0 (1.1)	0.001
Support from teachers (1–5)	2.2 (1.2)	1.8 (1.1)	0.007	2.0 (1.1)	1.8 (1.1)	0.023
DASS-21 Depression score	5.6 (4.2)	3.5 (4.1)	<0.001	4.9 (4.6)	3.3 (3.9)	<0.01
DASS-21 Anxiety score	5.7 (3.2)	3.9 (3.3)	<0.001	4.8 (3.3)	3.9 (3.4)	0.002
DASS-21 Stress score	8.7 (4.1)	5.9 (4.3)	<0.001	7.2 (4.1)	5.8 (4.4)	<0.01

DASS-21, Depression, anxiety, and stress scale—21 Items.

Table 1 also reveals that people who experienced cyberbullying showed significantly lower levels of attitude against cyberbullying (*p* = 0.022) and lower levels of perceived family (*p* < 0.001) and teachers support (*p* = 0.007), but a higher level of perceived peer support (*p* = 0.005) and higher depression (*p* < 0.001), anxiety (*p* < 0.001), and stress scores (*p* < 0.001) than did those not experiencing cyberbullying. These differences were also observed between participants with and without cyberbullying observation (*p* < 0.05), except for the level of cyberbullying attitude.

**Figure 1:**
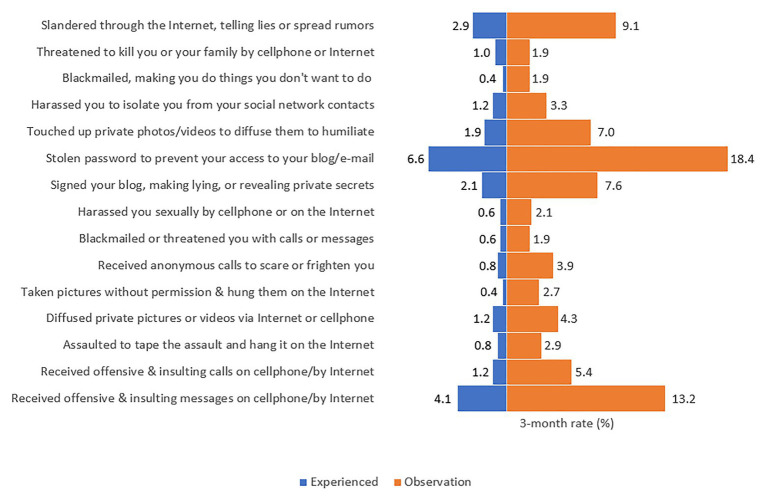
The 3-month rates of different cyberbullying behaviors in the study population (*n* = 484).

Figure 1 illustrates the 3-month rate of each cyberbullying behavior in terms of experience and observation. “Stole password to prevent access to blog/email” was the most common behavior when 6.6 and 18.4% of participants ever experienced and observed this behavior, respectively. “Received offensive & insulting messages on cellphone/by Internet” and “Slandered through the Internet, telling lies or spread rumors” were the second and third most common acts.

Among participants experiencing cyberbullying as victims, Table 2 indicates that 26.8% of students felt anxiety and fear and did not want to be close to anyone. Over 21% of victims had reduced study performance, 12.5% had suicidal ideation, and even 10.7% had suicidal attempts. However, only 48.2% tried to stop the perpetrators, and 35.7% told this experience to their friends. None of them told teachers, parents, or relatives. “Try to cope by myself,” “I think my parents, teachers or adults do not understand or believe me,” and “I think there’s nothing my parents, teachers or adults can do to help me” were the three most common reasons for not reporting cyberbullying acts among victims.

**Table 2:** Impacts, reactions, supporters, and reasons for not reporting cyberbullying experience (*n* = 56).

Characteristics	*n*	%
**Impacts**		
Decrease academic performance	12	21.4
Drop out of school	5	8.9
Start to substance use	2	3.6
Anxiety and fear	15	26.8
Not want to be close to anyone	15	26.8
Having suicidal ideation	7	12.5
Trying to self-harm or suicidal attempt	6	10.7
**Reactions against cyberbullying**		
None	4	7.1
Tell person performing online harassing, intimidating, or bullying to stop	27	48.2
Log out or leave cyberspace/not use Internet	19	33.9
Tell teachers, parents, or relatives	0	0.0
Tell your friends	20	35.7
Repeating cyberbullying behaviors against other people on the Internet	0	0.0
Performing bullying behaviors in real-life	0	0.0
**Reasons for not reporting cyberbullying experience**		
I think my parents, teachers, or adults do not understand or believe me.	8	14.3
I think there’s nothing my parents, teachers, or adults can do to help me.	8	14.3
If I tell my parents, teachers, or the adults, those who bully me online might get revenge and make things worse.	4	7.1
If I notify my parents, teachers, or adults, they may try to limit or prohibit me from accessing the Internet or other technology devices.	9	16.1
Others will laugh at me if I tell my parents, teachers, or adults.	4	7.1
I want to learn how to cope and deal with cyberbullying by myself.	11	19.6
There’s nothing serious about harassing, intimidating, or being bullied online. Everyone soon forgets, and no one will notice it anymore.	7	12.5

Meanwhile, among observers, 111 students (81%) were grouped into the “Intervene” category; 56 students (40.9%) and nine students (6.6%) were classified into “Ignore” and “Join in” categories, respectively (one student could do more than one act toward cyberbullying observation). Being afraid of having limited use of the Internet if they reported cyberbullying behaviors, thinking that adults did not understand or believe, and thinking that adults could not do anything for victims were the three most popular reasons for not reporting the cyberbullying behaviors among observers (Table 3).

**Table 3:** Reactions and reasons for not reporting among cyberbullying observation (*n* = 137).

Characteristics	*n*	%
Reactions^*^		
Intervene	111	81.0
Ignore	56	40.9
Join in	9	6.6
**Reasons for not reporting cyberbullying acts**		
I think my parents, teachers, or adults do not understand or believe me.	43	31.4
I think there’s nothing my parents, teachers, or adults can do to help victims.	34	24.8
I’m afraid of getting into trouble because I’m also at fault for my friends being bullied online.	19	13.9
I’m afraid of getting into trouble because people who bully my friends online might get revenge on me.	31	22.6
I am afraid that if my parents and adults know about cyber-violence, my parents will try to limit or prevent me from using phones, the Internet, or other technology devices.	46	33.6
There is nothing serious about being bullied online. Everyone will also quickly forget.	12	8.8
The fact that my friends are bullied on the Internet is not related to me, so I have no responsibility to report.	9	6.6

* Intervene included (1) “oppose acts of cyberbullying”; (2) “try to help or comfort the victim”; and (3) “report online violence to people able to help the victim (e.g., teachers and parents).” Ignore included “leaving cyberspace”; and Join in included “Encouraging cyberbullying behaviors” and “Enjoys cyberbullying acts, and wants to learn more, but does not participate or promote publicly.”

Table 4 shows associated factors with cyberbullying experience and observation. Cyberbullying experience was only found to be associated with cyberbullying observation (OR = 5.86, 95% *CI* = 3.06–11.21). Meanwhile, being female and of higher age were positively related to cyberbullying observation, whereas having a higher level of support from family (OR = 0.76, 95% *CI* = 0.61–0.94) was negatively associated with the cyberbullying observation.

**Table 4:** Associated factors with cyberbullying experience and observation.

Characteristics	Cyberbullying experience		Cyberbullying observation	
	OR	95% CI	OR	95% CI
Gender (female vs. male^a^)	0.63	0.34–1.18	1.95^**^	1.22–3.12
Age (vs. 11 years^a^)				
12 years	0.74	0.22–2.44	3.72^**^	1.61–8.58
13 years	0.91	0.38–2.17	5.10^**^	2.59–10.03
14 years	0.89	0.36–2.15	4.58^**^	2.31–9.08
Type of family (vs. nuclear^a^)				
Multiple generations	1.58	0.85–2.95	1.27	0.80–2.03
Others	1.35	0.15–12.42	1.19	0.25–5.62
Support from family (per score)	0.79	0.59–1.05	0.76^*^	0.61–0.94
Support from peers (per score)	1.24	0.89–1.72	1.19	0.94–1.51
Support from teachers (per score)	1.03	0.76–1.40	0.86	0.68–1.09
Cyberbullying attitude (per score)	0.93	0.80–1.09	1.00	0.87–1.15
Cyberbullying experience (yes vs. no^a^)			5.86^**^	3.05–11.25
Cyberbullying observation (yes vs. no^a^)	5.86^**^	3.06–11.21		

a Reference group.

* *p* < 0.05;

** *p* < 0.01.

In regression analysis, only cyberbullying attitude was found to be associated with “intervene” behaviors (OR = 1.53, 95% *CI* = 1.10–2.12). Participants having a higher level of peer support were more likely to ignore the cyberbullying acts (OR = 1.78, 95% *CI* = 1.08–2.93), while those having a higher level of teacher support were less likely to ignore these acts (OR = 0.64, 95% *CI* = 0.42–0.98; Table 5).

**Table 5:** Associated factors with different types of cyberbullying observation.

Characteristics	Intervene		Ignore	
	OR	95% CI	OR	95% CI
Gender (female vs. male^a^)	0.71	0.24–2.10	0.67	0.29–1.53
Age (vs. 11 years^a^)				
12 years	1.39	0.16–11.69	0.95	0.20–4.40
13 years	0.87	0.17–4.44	0.80	0.23–2.75
14 years	0.56	0.11–2.92	1.10	0.32–3.85
Type of family (vs. nuclear^a^)				
Multiple generations	1.14	0.42–3.13	0.96	0.44–2.12
Others			2.73	0.22–34.09
Support from family (per score)	0.94	0.59–1.48	0.72	0.50–1.05
Support from peers (per score)	0.97	0.55–1.69	1.78^*^	1.08–2.93
Support from teachers (per score)	0.99	0.61–1.61	0.64^*^	0.42–0.98
Cyberbullying attitude (per score)	1.53^*^	1.10–2.12	1.64	0.96–2.81
Cyberbullying experience (yes vs. no^a^)	1.07	0.34–3.31	0.81	0.34–1.94

a Reference group.

* *p* < 0.05.

**Figure 2:**
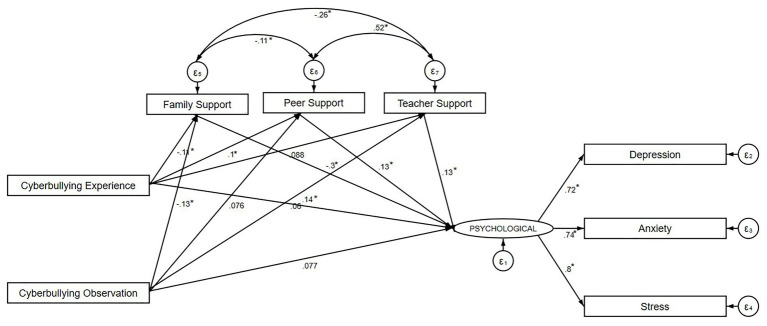
Mediation model of cyberbullying experience and observation on psychological health *via* social support. The model shows the standardized estimates for direct effects, the covariance between mediators, and dependent variables. ^*^p < 0.05.

The SEM is illustrated in Figure 2. The goodness-of-fit indices were acceptable with RMSEA = 0.052, CFI = 0.983, and SRMR = 0.026. The model shows that only cyberbullying experience showed a significantly positive direct effect on psychological problems. The cyberbullying experience group was significantly related to family and peer support, while the cyberbullying observation group was only significantly associated with family support. Only family support showed to be negatively associated with psychological problems, whereas peer and teacher support showed positive relations with psychological problems.

Table 6 determines the mediation or indirect effects of different types of support. For both cyberbullying experience and observation, only family support was found to partially mediate associations between cyberbullying experience and observation with levels of psychological problems among our participants. Regarding cyberbullying experience, the indirect effect of family support accounted for 18.3% of the total effect and 22.4% of the direct effect. Meanwhile, regarding cyberbullying observation, this indirect effect accounted for 32.5% of the total effect and 48.1% of the direct effect.

**Table 6:** Standardized indirect effects of cyberbullying experience and observation on psychological well-being outcomes *via* social support.

Pathways	Indirect effect	95% CI	Total effect/% total effect	Direct effect/% direct effect
Cyberbullying experience/family/psychological problems	0.032^*^	0.003/0.060	0.173/18.3%	0.142/22.4%
Cyberbullying experience/peer/psychological problems	0.014	−0.003/0.031	0.156/9.0%	0.142/9.9%
Cyberbullying experience/school/psychological problems	0.012	−0.0040.027	0.153/8.0%	0.142/8.0%
Cyberbullying observation/family/psychological problems	0.037^*^	0.008/0.066	0.114/32.5%	0.077/48.1%
Cyberbullying observation/peer/psychological problems	0.010	−0.005/0.025	0.087/11.7%	0.077/13.2%
Cyberbullying observation/school/psychological problems	0.008	−0.006/0.022	0.085/9.2%	0.077/10.2%

* *p* < 0.05.

## Discussion

Our study contributed to the current literature about the experience and coping strategies of adolescents toward cyberbullying and the potential effects of different support to mitigate the psychological consequence of cyberbullying. Our study suggested a low rate of cyberbullying experience and observation in secondary school students, as well as a high risk of psychological problems among those experiencing cyberbullying. Moreover, family support was an important protective source and helped mediate the relationship between cyberbullying and psychological problems.

The rate of adolescents involving in cyberbullying as victims over the past 3 months in our study was low, according to the self-report information from the participants. This rate was much lower than previous research in Hanoi, Vietnam, which showed that 45.1% of adolescents aged 13–18 were victims of cyberbullying in the past 12 months (Chi et al., 2020). Our result was equivalent to a previous study in the United States with 11% of adolescents who studied grades 6 to 8 being bullied online in the past 2 months (Kowalski and Limber, 2007). Overall, the estimated prevalence of cyberbullying varied significantly between different studies and countries due to the difference of cyberbullying definition, time frame, and methods of measurement (Berne et al., 2013; Selkie et al., 2016; Brochado et al., 2017), which results in the problematic study comparisons. Indeed, using the Cyberbullying Test, which was a valid scale to measure cyberbullying (Garaigordobil, 2017), was advantageous to our study compared with other prior research. This measure contains 15 cyberbullying behaviors, enabling us to capture more comprehensive aspects that participants might suffer or observe during the recall period than other previous studies (Hellfeldt et al., 2019; Livazović and Ham, 2019; Chi et al., 2020; Panumaporn et al., 2020).

Notably, our results showed that though the impact of cyberbullying was significant, the proportion of participants taking specific actions against cyberbullying was not high. In addition to the impact on academic performance, there was a high proportion of individuals who experienced cyberbullying suffering the negative psychological effects such as anxiety, fear, and even suicidal thoughts. This was similar to previous studies showing the serious consequences of cyberbullying on the lives of victims (Schneider et al., 2012; Nixon, 2014). Nonetheless, the findings showed that only nearly 50% of the victims tried to stop this act of the perpetration, and 33.9% tried to leave cyberspace. We also observed that victims only shared with friends about the issue of cyberbullying but did not share it with parents or teachers. The most common reasons for not sharing with parents or teachers were because the adolescents were afraid that the adults did not believe or the adults will not be able to do anything for the adolescents. Another reason was that the victims wanted to solve this problem themselves. In a previous study in Vietnam, it was found that ignoring this behavior and blocking the perpetrator’s account were the two most common measures (58.8 and 54.6%, respectively; Chi et al., 2020). However, problems caused by cyberbullying were not easy to deal with because these behaviors can occur at any time and can reach large numbers of audiences in a short period of time (Patchin and Hinduja, 2006), causing a feeling of stigma and isolation among victims (Bossler et al., 2012; Burton et al., 2013) and, in turn, worsening the consequences of cyberbullying. Therefore, equipping adolescents with appropriate coping strategies against cyberbullying is essential and should be prioritized in school and family.

Our study also examined the rate of cyberbullying observers among adolescents and identified how they reacted to cyberbullying behaviors. As expected, we found that the majority of observers were in the group “Intervene” who responded to the cyberbullying acts by reporting these behaviors or helping victims. This result was similar to the study in Thailand showing that most observers belonged to the “Intervene” group (Panumaporn et al., 2020). However, our study was different from study results in Belgium, where the authors show that most adolescents belonged to the “Ignore” group when observing cyberbullying behaviors (Van Cleemput et al., 2014). We assumed that the cultural factors might be attributable to this difference. While the Western culture highlighted individualism, the Asian culture emphasized the role of collectivism, which might motivate them to help others when they faced problems (Sittichai and Smith, 2015). On the other hand, similar to the victim group, the main reasons that participants in the observer group did not report violent cyber behavior included (1) being afraid of limited Internet use and (2) believing that adults were unable to address this issue. This result suggested a huge gap in the relationships between parent-teacher and adolescents, especially among cyberbullying victims or observers. Interestingly, results of the multivariate models indicated an opposite trend between peer and teacher support, when higher peer and teacher support were associated with higher and lower likelihoods of being “Ignore” people. This phenomenon could be justified that adolescents’ peers might not perceive this issue as a problem as adults did (i.e., parents and teachers; Slonje and Smith, 2008). Moreover, even when youth perceived the impacts of cyberbullying, they could not provide adequate support due to the lack of knowledge and resource needed. Therefore, instead of recommending interventions on the issue, they were more likely to advise to ignore cyberbullying behaviors. It should be noted that the attitude against cyberbullying played an important role among adolescents in the “Intervene” group. This finding was consistent with previous studies on both cyberbullying and traditional bullying (Yang and Kim, 2017; Panumaporn et al., 2020). Therefore, campaigns to motivate adolescents to intervene in cyberbullying are critical to diminish this behavior and its impacts.

The findings of this study echoed previous evidence showing that cyberbullying experience was associated with psychological problems (Beckman et al., 2012; Schneider et al., 2012; Nixon, 2014; Wong et al., 2014; Panumaporn et al., 2020). Moreover, our study underlined the protective mediation effects of family support on the association between cyberbullying experience and observation with psychological issues. Unlike traditional bullying where parent, friend, and teacher support helped to mitigate the impact of bullying acts on victims’ lives and mental health (Price and Dalgleish, 2010; Fanti et al., 2012; Hellfeldt et al., 2019), in our study, friend and teacher support did not buffer against the psychological problems among cyberbullying victims and/or observers. In literature, adolescents are concerned that sharing their experience with the teacher was an ineffective strategy (Price and Dalgleish, 2010), and reporting the problem to their parents could hinder their freedom in Internet use (Hoff Dianne and Mitchell Sidney, 2009). However, in a previous survey in Sweden, parent and teacher support was found to buffer against depressive and anxiety symptoms in cyberbullying victims and bully victims (Hellfeldt et al., 2019). Another longitudinal study found that adolescents who were victims of cyberbullying had lower levels of depressive symptoms if they had family support (Machmutow et al., 2012). As discussed above, we believed that parents could offer appropriate emotional support to help adolescents in controlling the psychological distress caused by cyberbullying behaviors (Livazović and Ham, 2019). Moreover, they may be more likely to perceive accurately the problem of cyberbullying, while friends might not fully recognize the trouble of cyberbullying involvement, resulting in the provision of inadequate support (Slonje and Smith, 2008). This result is critical since most of the victims in our study sought help from friends rather than their parents. Thus, parents should be proactive in building a strong bond with their children (Nixon, 2014; Slonje et al., 2017). On the other hand, our finding partially affirmed that peer support might not be the best focus to address cyberbullying and its consequence (Hellfeldt et al., 2019). However, further longitudinal studies should be performed to test the effect of peer support on buffering the relationship between cyberbullying and psychological impairment in different contexts.

The current study indicated several implications. First, educational campaigns should be performed to raise adolescents, parents, and teachers’ awareness and attitude toward cyberbullying, motivating them to become involved to intervene and prevent cyberbullying behaviors. The contents of these interventions should include knowledge and practices on cyberbullying, communication and Internet use skills, and prosocial behaviors, empathy, and coping strategies with cyberbullying (Hutson et al., 2018). Regular training sessions should be performed to help adolescents in acquiring skills and abilities to actively cope with cyberbullying, help other victims, and prevent them from joining in cyberbullying. Previous studies found that active strategies to cope with cyberbullying victimization were effective to address the cyberbullying-related issues (Ybarra et al., 2007; Riebel et al., 2009; Chan and Wong, 2017). Second, according to the study, leaders in schools and communities should implement activities that increase parents’ roles in addressing cyberbullying and its consequences among adolescents. Positive parent-children relationships could encourage adolescents to find support when dealing with difficult situations (Chan and Chui, 2015; Chan and Wong, 2017). Finally, given that cyberbullying is an emerging problem but school regulations for this issue do not exist in Vietnam, current policies should consider this type of bullying and its impact, especially among adolescents.

Interpretation of the study results should be done cautiously with the following limitations. First, given that cyberbullying is a sensitive issue, relying on only self-reports from participants may underestimate the actual rates of cyberbullying in adolescents. In literature, approaches that used information from multiple sources such as peers and teachers would be recommended (Brochado et al., 2017). Further studies that involve multiple reporters to measure the prevalence of cyberbullying should be taken into account. Second, we used the cross-sectional design, which limited our ability to establish the causal associations. Thus, conclusions about the associations and effects in this study cannot be definitely drawn. Future studies should use longitudinal designs to investigate the influence of cyberbullying and social support on psychological well-being in middle school adolescents. Third, the rate of cyberbullying experience and observation was low, resulting in a small sample size for statistical tests. Moreover, we used a modified instrument to measure cyberbullying experience rather than use the original one, which might underestimate or overestimate the rate of cyberbullying victimization and observation. Finally, other characteristics such as traditional bullying exposure, the Internet or social media use, and cyberbullying perpetration were not fully investigated. These factors were found to be associated with cyberbullying victimization in previous work (Beckman et al., 2012; Athanasiou et al., 2018; Chi et al., 2020). Hence, further studies should be elucidated to measure these relationships between these factors and cyberbullying experience.

## Conclusion

Our study suggested that the 3-month rate of cyberbullying experience and observation among urban adolescents aged 11–14 in Vietnam was low. However, current coping strategies against cyberbullying in this group were not sufficient. Family support is an important factor that should be considered for designing interventions to mitigate the impacts of cyberbullying on the mental health of adolescents.

## Data Availability Statement

The raw data supporting the conclusions of this article will be made available by the authors, without undue reservation.

## Ethics Statement

The studies involving human participants were reviewed and approved by Hanoi Medical University. Written informed consent to participate in this study was provided by the participants’ legal guardian/next of kin.

## Author Contributions

AN, AT, BT, LN, MH, and TrN: conceptualization. AN, AT, BT, TrN, LD, and GV: methodology. AN, AT, LN, MH, and GV: formal analysis and investigation. AN, AT, BT, LN, MH, TrN, LD, GV, TuN, HD, CL, RH, and CH: writing—original draft preparation, and review and editing. BT, CL, RH, and CH: supervision. All authors contributed to the article and approved the submitted version.

### Conflict of Interest

The authors declare that the research was conducted in the absence of any commercial or financial relationships that could be construed as a potential conflict of interest.
